# Is Biofeedback for Vertigo Effective in Ordinary Medical Centers? A Controlled Trial in Northern Italy

**DOI:** 10.1007/s10484-023-09588-0

**Published:** 2023-05-25

**Authors:** Chiara Buizza, Elena Franco, Alberto Ghilardi

**Affiliations:** 1 Department of Clinical and Experimental Sciences, University of Brescia, Viale Europa 11, Brescia, Italy; 2 Medical Center San Francesco, Via Zadei 16, Brescia, Italy

**Keywords:** Vertigo, Biofeedback, Effectiveness, Disability

## Abstract

The aim of this study was to assess the therapeutic effectiveness of biofeedback, in a medical center’s routine for treating vestibular disorders, reducing emotional, functional, and physical disability at three-month follow-up. A total of 197 outpatients were recruited from a medical center to treat vestibular disorders. Patients in the control group received treatment as usual, consisting of one monthly visit with an otolaryngologist and pharmacological treatment specific for vertigo, while the experimental group attended biofeedback training. Patients in the experimental group received pharmacological therapy only in the phase before the start of biofeedback in order to stabilize the acute phase. During the three-month follow-up, the experimental group did not receive any booster sessions of biofeedback. At three-month follow-up there was a statistically significant difference between the groups, both in the mean total score of the dizziness handicap inventory and in the three subscales: physical, emotional, and functional. Moreover, the biofeedback group had reduced psycho-physiological parameters for all average values at three-month follow-up compared to the baseline. This is one of few studies assessing the effectiveness of biofeedback in a naturalistic setting for vestibular disorder treatment. The data confirmed that biofeedback can impact illness course, in terms of self-perceived disability reduction, assessed on emotional, functional, and physical aspects of daily living.

## Introduction

Vertigo affects about 30% of the population (Neuhauser, 2007). Its prevalence increases with age and its incidence is estimated to be three times higher in women than in men (Bronstein, 2013). Vertigo is a particular sensation caused by altered cooperation between different organs which inform the brain of the position of the body in space and its state of rest or movement (Filipo, 1973). Vertigo is therefore the interpretation of uncoordinated stimuli arriving at higher centers, resulting in a sensation of movement, while the other sense organs indicate that the body is in a state of rest or vice versa. These illusory sensations are defined respectively as subjective vertigo and objective vertigo. By subjective vertigo, we mean that sensation for which patients have the illusion of moving their body in space, while objective vertigo indicates the sensation of displacement of the environment (Filipo, 1973). Vertigo symptoms have a multifactorial cause: they can be caused by diseases limited to the maculo-ampullary apparatus of the labyrinth (labyrinthine disorders), or by peripheral or central nervous diseases, as well as being triggered by stressful situations. Vertigo can last for between a few seconds to several days, with episodes that can recur more or less frequently. The vertiginous sensation that is often accompanied by neuro-vegetative disorders such as nausea, vomiting, headaches, and sweating causes a situation of stress, anxiety, and fear, and when it persists for a long time, also depression. This causes consequent impairment of psycho-social functioning of those afflicted (Bayat et al., 2020; Neuhauser et al., 2008). It is therefore a complex pathology that not only affects the ear, but can also affect other parts of the body and for this reason requires multi-disciplinary treatment involving several professional figures. Therapeutic strategies also involve various levels of complexity and can be provided at home or in both outpatient and inpatient settings, depending on the severity of the disease (Edlow et al., 2018; Libonati, 2016; Newman-Toker et al., 2013; Weber et al., 2009). A prognosis of vertigo is very complex and variable. Indeed, some patients show an improvement in vertigo symptoms within six months, while in others, resolution is incomplete with persistent resistance to typical medical therapy and recurrent episodes of vertigo (Bronstein et al., 2010; Luxon et al., 2004). As underlined by current scientific research, the most frequent causes of this resistance to medical therapy usually involve psychological problems that are often associated with the consequences of vestibular pathologies (Beidel et al., 2001; Luxon et al., 1997).

**Fig. 1:**
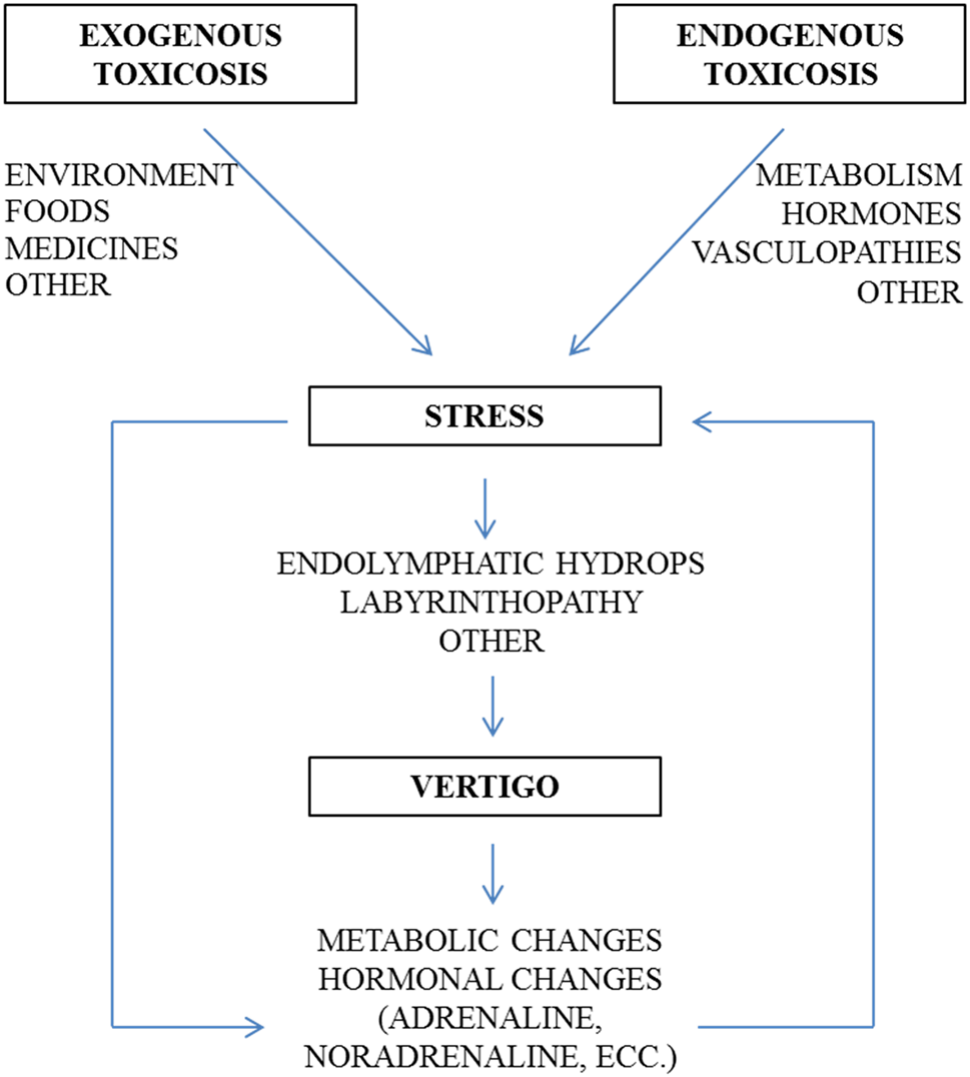
Mechanisms underlying the etiology of balance disorders (based on Saman et al., 2012)

Until now, the treatment of vertigo has almost always been of a medical nature, with a privileged focus on organic therapies. Few are the psychological treatment regimes proposed. Among these, biofeedback can possibly be an effective technique given that has long been used for stress management (Goessl et al., 2017; Hallman et al., 2013; Lehrer et al., 2013; MacKinnon et al., 2013; Ratanasiripong et al., 2015; Schwartz & Andrasik, 2017; Sherlin et al., 2009), and considerably so for tinnitus (Bardsiri et al., 2018; Bauer, 2018; Heinecke et al., 2009; Henry et al., 2005; Hesser et al., 2011; House, 1978; Meckley Kutyana et al., 2015; Weise et al., 2008). Indeed, cochlear hydrops at the base of tinnitus has an etiology similar to endolymphatic hydrops, which is the basis of vertigo. Figure 1 shows the mechanisms underlying the etiology of balance disorders, in which there is a vicious circle generated by stress conditions, which in turn are the cause of balance disorders and a consequence for the disability of this pathology.

The few studies available on the treatment of vestibular disorders through biofeedback have shown that it reduces the symptoms of vertigo, anxiety, and depression (Liu et al., 2017), and improves motor symptoms by helping patients to acquire information about postural changes in their body (Necdet Ardıç et al., 2021). However, these studies are based on small samples and have not had any follow-up studies. Indeed, we know that vestibular disorders can occur over time with cyclicality and chronicity (Staab et al., 2017).

This study has measured the therapeutic effectiveness of biofeedback in the routine of a medical center to treat vestibular disorders and assessed its efficacy in reducing emotional, functional, and physical disability during three-month follow-up.

## Materials and Methods

### Study Design

This controlled study involved two groups of outpatients: patients in the control group received treatment as usual (TAU), consisting of one monthly visit with a treating otolaryngologist and pharmacological treatment specific for vertigo (i.e., anti-vertigo, corticosteroid, prokinetic, diuretic medications), while the experimental group attended biofeedback training. Patients in the experimental group had pharmacological therapy only in the phase immediately before the start of biofeedback, in order to stabilize the acute phase, and during biofeedback they had none. During the three-month follow-up, the experimental group did not receive any biofeedback boosting sessions.

### Participants

All otolaryngologists working at the medical center were informed that they could refer patients with vestibular disorders for biofeedback treatment. All referred patients had to meet the following inclusion criteria: diagnosis of labyrinthine endolymphatic hydrops or vascular labyrinthopathy; aged > 18 years; pharmacological stabilization of the acute phase for autonomic symptoms (e.g., nausea and vomiting) and vestibular symptoms that prevent biofeedback treatment; had adequate native language skills to complete the questionnaire. Exclusion criteria were vertigo symptoms caused by dementia or substance abuse. One hundred and ninety-seven patients with vertigo, aged 18–85 years, were consecutively recruited from May 2019 to May 2020.

The study was a pragmatic trial conducted under routine conditions and therefore, randomization was not possible. Moreover, for ethical reasons, we could not withdraw eligible patients from a potentially beneficial intervention. In an attempt to overcome this problem, we opted to use a natural control group, which consisted of patients selected for participation, but kept on a waiting list due to space limitations. Indeed, given the limited number of biofeedback therapists, only 106 patients could be treated at the medical center over the course of one year. As a result, the only patient selection and participation criterion was temporal priority.

The study was conducted according to the principles outlined in the declaration of Helsinki and approved by the management of the Vertigo Treatment Center. All participants signed a written informed consent prior to the start the study and were free to leave the study at any time.

### Biofeedback Group

The biofeedback treatment consisted of minimum 1 to maximum 5 monthly sessions. Each session lasted approximately 60 min. During training, we used session biofeedback to regulate respiratory frequency, heart rate, and muscle tension parameters, and we analyzed cognitive, emotional, psychological factors associated with vertigo. Between sessions, patients were asked to do exercises to improve breathing, relax musculature, and manage the pathology.

All psycho-physiological data were recorded using Procomp Infinity by Thought Technology Ltd., an eight-channel computer-operated encoder. The software used was BioGraph Infinitiy (version 6.2.0). Muscle activity was assessed for the forehead (frontalis region), jaws (masseter, bilateral), and neck (sternocleidomastoids, bilateral). Particularly important for patients with vertigo, EMG electrodes were placed over the anterolateral muscles of the sternocleidomastoid neck. According to recommendations by Cram et al. (1990), the electrodes were placed parallel to muscle fibers to maximize sensitivity and selectivity. We used T9503M MyoScan EMG sensors, pre-amplified, with input impedance greater than 10 GW and an active range from 10 to 500 Hz, according to Cacioppo et al. (2000). We used dry T340M Triodes as electrodes. The raw EMG signal was converted to root mean square (RMS) using the non-sliding-window algorithm with an averaging factor of 10 and a time period of 1s. The artefact was tolerated to ensure measurement of slow muscle activity. The skin conductance level (SCL) was then recorded by SA9309M Skin Conductance Flex/Pro with a signal range from 0 to 30 lS. Finger temperature was measured with a SA9310M sensor, signal ranging from 12.5 to 40.5 °C. The skin was cleaned with an antiseptic before attaching the triodes. The respiratory and heart rate were measured with a breath sensor SA9311M and BVP frequency sensor and plethysmography wrist band SA9308M.

### Standardized Assessment

All patients were assessed using the Dizziness Handicap Inventory (DHI). This is a self-report questionnaire to evaluate the effect that dizziness and unsteadiness have on emotional, functional, and physical aspects of daily living (Jacobson et al., 1991; Jacobson & Newman, 1990; Newman et al., 1993; Newman & Jacobson, 1993). The instrument consists of 25 items with 3 possible answers: *yes* (4 points), *sometimes* (2 points), *no* (0 points). The total score ranges from 0 (no disability) to 100 (maximum self-perceived disability), with a cut-off ≥ 31.

The Italian version of the DHI is a reliable instrument, as indicated by a Cronbach’s alpha of 0.92 for the DHI total score, and of 0.82, 0.84, and 0.75 for the functional, emotional, and physical sub-scales, respectively (Nola et al., 2010). Participants were also requested to fill out a socio-demographic and clinical form to collect information such as sex, age, employment status, diagnosis, symptoms onset, previous hospitalizations, and familiarity for vertigo. Moreover, psycho-physiological parameters (EMG, respiratory rate, skin conductance, peripheral temperature, heart rate, lf/hf) were all assessed during DHI administration with an initial and final baseline of 2 min for the experimental group, who attended biofeedback training.

We decided to use biofeedback to voluntarily alter physiological parameters, respiratory rate in particular, because of the correlation between nystagmus and increased respiratory variability (Edlow et al., 2018; Libonati, 2016; Newman-Toker et al., 2013; Weber et al., 2009). Nystagmus is the clinical indicator of the vertiginous syndrome (Johkura, 2013; Monday & Tétreault, 1980; Park et al., 2011; Post & Dickerson, 2010). It has been hypothesized that the self-regulation of sympathetic arousal, in particular respiratory self-regulation through biofeedback, could reduce hyperventilation. Furthermore, the reduction in breathing rates with biofeedback leads to the disappearance of nystagmus and, as a consequence, also of vertigo (Stahl et al., 2002). The experimental group underwent respiratory biofeedback according to the following indications (Khazan, 2013).

1. Explain the patient the physiology of breathing and overbreathing.
2. Teach awareness of breathing, helping the patient pay attention to the emotions associated with effortless breathing.
3. Teach slow and deep diaphragmatic breathing without feedback.
4. Teach controlled diaphragmatic breathing with a ratio of 40:60 from inhalation to exhalation, with and without a pause between an exhalation and the next inhalation. In particular, the resonance frequency of the respiratory frequency is determined and diaphragmatic breathing is trained to the rhythm of the resonant frequency. During the training sessions, patients were taught to increase HRV according to the available protocols, in particular the protocol for Heart Rate Variability Biofeedback Training (Lehrer et al., 2013). During the sessions it was possible to teach the patients to breathe at a resonance frequency of the cardiovascular system. Indeed, at this frequency, respiratory effects on heart rate stimulate baroreflex effects, such that both respiratory sinus arrhythmia and baroreflex gain are maximized (Lehrer et al., 2013). Also, breathing at this frequency causes heart rate to go up and down in phase with respiration (heart rate increases with inhalation, decreases with exhalation) and respiratory gas exchange efficiency is maximized (Vaschillo et al., 2004; Yasuma & Hayano, 2004). Patients are able to produce very large increases in HRV through biofeedback because of ‘resonance’ characteristics in the cardiovascular system. HRV biofeedback stimulates a particular reflex in the cardiovascular system, called ‘baroreflex’ and it helps to control blood pressure. It also helps to control emotional reactivity and promotes breathing efficiency. When blood pressure goes up, the baroreflex causes heart rate to go down, and when blood pressure goes down, heart rate goes up (Lehrer, 2013; Vaschillo et al., 2006). This causes a rhythm in heart rate fluctuations, and when patients breathes at this exact rhythm, the system resonates (Lehrer, 2013; Vaschillo et al., 2006). When people breathe at this frequency, the baroreflex system is stimulated and strengthened (Lehrer et al., 2003), and through projections to other systems in the body (e.g., inflammatory and limbic systems), other events occur that produce the many beneficial effects of HRV biofeedback.
5. Practice breathing at home, for 20 min twice a day, by training breathing with the second hand of a clock or resonant-frequency breathing rhythm downloaded from a computer; instruct the patient to be aware of symptoms of hyperventilation and, if it occurs, to breathe more shallowly and naturally.

Furthermore, the experimental group underwent skin conductance biofeedback and EMG biofeedback according to the following indications (Khazan, 2013): (1) teach progressive muscle relaxation without feedback; (2) introduce biofeedback using visual and audible information referenced to skin conductance and EMG muscle tension benchmarks; (3) monitor skin conductance and muscle tension during Jacobson’s progressive muscle relaxation and monitor the change in response; (4) assign muscle relaxation exercises to be performed at home and improve awareness.

### Three-Month Follow-Up

Three months after the end of the last biofeedback session, a follow-up was scheduled for psychophysiological re-evaluation. During follow-up, the participants assigned to the biofeedback group, who had already attended their treatment sessions, were monitored with EMG, respiratory rate, skin conductance, peripheral temperature, heart rate, and lf/hf parameters, during DHI administration and with an initial and final baseline of two minutes. Three-month follow-up was also performed for the control group. In this case, only the DHI was re-administered.

### Main Outcome Measurement

Our main outcome was to assess the effectiveness of biofeedback in an ordinary medical center for treating vestibular disorders. This was considered in terms of self-perceived disability reduction, assessed on emotional, functional, and physical aspects of daily living, through the DHI questionnaire (score < 31) at 3-month follow-up.

### Statistical Analysis

Descriptive statistics for socio-demographic and clinical characteristics are given in terms of mean and standard deviation for numerical variables and percentage distribution for categorical ones. The t-test or the corresponding non-parametric Mann–Whitney test (for non-Gaussian distributed variables) was used to compare differences of quantitative variables between two groups and to compare any differences within the experimental group attending biofeedback training. The magnitude of the effects is reported using Coehn’s d or standardized mean difference, for independent samples. In our analysis, 0.2 was considered a small effect, up to 0.5 a medium effect, and 0.8 or above a large effect. The Chi-square test was used to compare categorical variables between groups. We calculated the adjusted residuals (z-scores) and their associated p values to detect any positive (z > 1.96) and significant (p < 0.05) relationships between the variables. All tests were two-tailed and the probability of type I error was set at p < 0.05. All analyses were performed with SPSS 26.

## Results

### The Groups’ Baseline Characteristics

Both groups were comparable at baseline. Table 1 shows the main characteristics of the two samples. The experimental group consisted of 106 patients (34 men and 72 women); the control group consisted of 91 participants (26 men and 65 women). There were no statistically significant differences observed for the participants’ main characteristics, with the exception of age: biofeedback group had a higher average age (M = 56.7, SD = 17.2) than controls (M = 49.0, SD = 18.9; t = 2.856, *p* = 0.005). The effect size was small (η^2^ = 0.04). The main diagnosis in both groups was labyrinthine endolymphatic hydrops (90.6% in the biofeedback group versus 87.9% in the control group). There were no between-group differences for previous hospitalization (30.2% in the biofeedback group versus 33.0% in the control group), or for familiarity with vertigo (26.4% in the biofeedback group *versus* 28.6% in the control group). Furthermore, there were no differences between the two groups in terms of pharmacological treatment (see Table 1).

**Table 1:** Comparison of sociodemographic and clinical characteristics of participants at baseline

	Biofeedback group (N = 106)	Controls (N = 91)	Statistical test	p-value
Age, mean (SD)	56.7 (17.2)	49.0 (18.9)	3727.5	0.005^a^
Illness duration, mean (SD)	5.8 (7.8)	4.6 (4.9)	4875.5	0.89^b^
Sex, n (%)				
Male	34 (32.1)	26 (28.6)	0.284	0.59^c^
Female	72 (67.9)	65 (71.4)		
Employed, n (%)				
Yes	56 (52.8)	57 (62.6)	1.925	0.16^c^
No	50 (47.2)	34 (37.4)		
Diagnosis, n (%)				
Labyrinthine endolymphatic hydrops	96 (90.6)	80 (87.9)	0.362	0.54^c^
Vascular labyrinthopathy	10 (9.4)	11 (12.1)		
Previous hospitalizations, n (%)				
Yes	32 (30.2)	30 (33.0)	0.175	0.67^c^
No	74 (69.8)	61 (67.0)		
Familiarity for vertigo, n (%)				
Yes	28 (26.4)	26 (28.6)	0.114	0.73^c^
No	78 (73.6)	65 (71.4)		
Drugs therapy, n (%)				
Antivertigo				
Yes	93 (87.7)	83 (91.2)	0.620	0.43^c^
No	13 (12.3)	8 (8.8)		
Corticosteroid				
Yes	23 (21.7)	19 (20.9)	0.020	0.88^c^
No	83 (78.3)	72 (79.1)		
Prokinetic				
Yes	106 (100)	91 (100)	–	–
No	0 (0.0)	0 (0.0)		
Diuretic				
Yes	12 (11.3)	10 (11.0)	0.005	0.94^c^
No	94 (88.7)	81 (89.0)		
DHI, mean (SD)				
Total score	50.4 (18.7)	50.4 (18.2)	4741.1	0.83^b^
Physical score	13.9 (6.8)	15.2 (7.0)	5331.5	0.19^b^
Emotional score	17.3 (8.2)	17.4 (8.5)	8960.0	0.90^b^
Functional score	19.8 (8.1)	17.6 (7.6)	4103.0	0.06^b^

^a^T-test

^b^Mann-Whitney

^c^Chi square; df = 1

The total DHI mean score was comparable between the groups and showed a high degree of self-perceived disability (score ≥ 31). The total mean score was 50.4 (SD = 18.7) in the biofeedback group and 50.4 (SD = 18.2) in the control group. The most affected aspects in both groups were the emotional and functional ones.

### Differences Between Groups at Three-Month Follow-Up

At three-month follow-up, there was a statistically significant difference between groups in the total score of DHI (Table 2.). The total mean score was 19.1 (SD = 11.0) in the biofeedback group and 43.8 (SD = 20.2) in the control group (U = 8.455, p < 0.0001). The effect size was medium (η^2^ = 0.4). Furthermore, in the three DHI sub-scales, the biofeedback group had a significant reduction compared to controls. The mean physical score was 7.9 (SD = 5.2) in the biofeedback group and 11.9 (SD = 6.5) in the control group (U = 6.560, p < 0.0001, η^2^ = 0.1). The mean emotional score was 3.1 (SD = 4.4) in the biofeedback group and 15.4 (SD = 9.7) in the control group (U = 8.656, p < 0.0001, η^2^ = 0.5). The mean functional score was 8.1 (SD = 5.3) in the biofeedback group and 16.5 (SD = 7.5) in the control group (U = 7.874, p < 0.0001, η^2^ = 0.3).

**Table 2.** Differences between groups at 3-month follow-up

	Biofeedback group (N = 106)	Controls (N = 91)	Statistical test	p-value
DHI, mean (SD)				
Total score	19.1 (11.0)	43.8 (20.2)	8.455	< .0001^a^
Physical score	7.9 (5.2)	11.9 (6.5)	6.560	< .0001^a^
Emotional score	3.1 (4.4)	15.4 (9.7)	8.656	< .0001^a^
Functional score	8.1 (5.3)	16.5 (7.5)	7.874	< .0001^a^

^a^Mann–Whitney

### Differences Between Baseline and Three-Month Follow-Up in the Biofeedback Group

Patients followed an average of 2.3 (SD = 1.3) biofeedback sessions. No patients discontinued their treatment. Table 3 shows the differences between baseline and three-month follow-up in psycho-physiological parameters in the biofeedback group. There was a reduction of psycho-physiological parameters for all average values at three-month follow-up compared to the baseline. At baseline, the average psycho-physiological parameters were 99.2 µV for EMG, 2.1 µS for skin conductance and 74 bpm for BVP heart rate. At three-month follow-up, there was an average value of 26.8 µV for the EMG parameter, 1.4 µS for the skin conductance and 68.6 bpm for the BVP heart rate was obtained (p value < 0.0001 for all measured parameters). At baseline, a mean maximal respiratory rate of 19.4 breaths per minute and a mean maximal heart rate of 89.9 bpm were observed with biofeedback. At three-month follow-up, a mean maximal respiratory rate of 15.6 breaths per minute and a mean maximal heart rate of 81.5 bpm (p value < 0.0001 for all measured parameters) were observed. With biofeedback training patients were able to produce very large increases in HRV because of ‘resonance’ characteristics in the cardiovascular system since HRV biofeedback stimulates a particular reflex in the cardiovascular system, called ‘baroreflex’ which helps control blood pressure.

**Table 3:** Differences in psychophysiological parameters within biofeedback group

Psychophysiological parameters	Baseline mean (SD)	Follow-up mean (SD)	Test Wilcoxon	*p* value
EMG	99.2 (147.1)	26.8 (76.2)	− 6.155	< 0.0001
Skin conductance	2.1 (1.8)	1.4 (2.0)	− 4.944	< 0.0001
Peripheral temperature	31.4 (3.3)	30.4 (3.8)	− 2.897	0.004
Elevations respiratory rate	19.4 (1.0)	15.6 (81.5)	− 8.747	< 0.0001
Elevations BVP	89.9 (11.7)	81.5 (62.2)	− 8.135	< 0.0001

### Dose–Response Effect

**Fig. 2:**
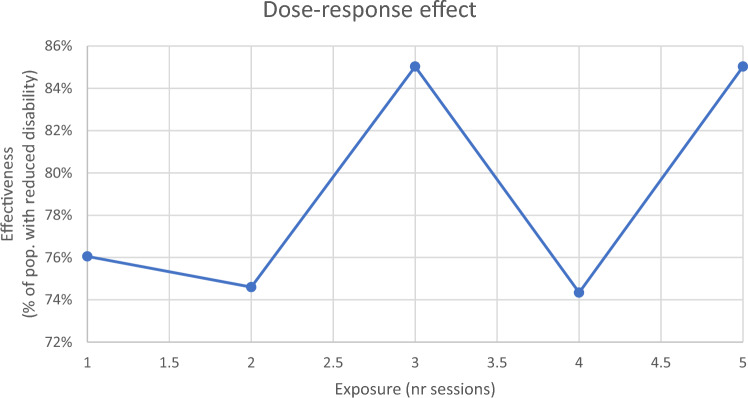
Dose–response effect

Figure 2 shows the dose–response effect. The graph represents the dose–response analysis, depicting the impact of symptom reduction for the sample target (response) after each session (dose). The process started creating dummy variables for the main outcome (DHI), taking the cut-off (31) as a reference, coding with 1 score below the cut-off (corresponding to a ‘response’), and with a 0 score above the cut-off (corresponding t to no ‘response’). This was done for both the baseline and three-month follow-up measurements. Only the delta was taken as a reference, meaning that only a positive change from baseline to the three-month follow-up was considered an actual treatment ‘response’. The ratio of patients giving a response over the total number of patients with the condition, corrected for patients giving a response and not part of the intervention (controls), was transformed into a percentage and is shown on the Y axis. We can clearly see the effectiveness for each set of sessions as being above 70%. A Probit regression was finally performed in order to obtain the ED50 (median effective dose, i.e., the dose necessary for an impact of 50% of the exposed sample), which corresponds to 0.013 sessions. One session is more than enough for a significant change for individuals, in terms of reduction of symptoms of the perceived disability associated with dizziness.

## Discussion

This study aimed to assess the therapeutic effectiveness of biofeedback in the routine of a medical center for treating vestibular disorders, in reducing emotional, functional, and physical disability during three-month follow-up. Few are the studies currently available on the treatment of vertigo using biofeedback (Liu et al., 2017; Necdet Ardıç et al., 2021). These studies, mainly based on vestibular rehabilitation, show the effectiveness of biofeedback in improving the quality of life of patients suffering from vertigo. However, they do have several limitations, in particular, the sample’s small size, the biofeedback group followed mixed treatment (biofeedback plus drugs), and the follow-up was very short. In our study, the experimental group was treated exclusively with biofeedback, allowing us to consider the impact of biofeedback compared with pharmacological treatment. Moreover, the longer follow-up, considered the cyclical nature of the disorder and the possible recurrence of vestibular disorders (Staab et al., 2017), allowed us to monitor any benefits produced by biofeedback treatment over time.

This study showed that both groups had a high level of self-perceived disability at baseline. In particular, functional and emotional aspects were the most compromised, showing the impact of vertigo on the daily life of these patients. The DHI functional aspects considered the interference of dizziness on the performance of certain eye, head, and body movements, although focusing on the capacity of performing professional, domestic, social, and leisure activities as well as on the independency in performing tasks such as walking without help and walking around the house in the dark. The emotional aspects investigated the possible harm caused by dizziness on the quality of life, which can generate frustration, fear of going outside without company or staying at home alone, shame regarding clinical manifestations, worries about concentration disorders, a sensation of incapacity, changes in family or social relationships, and depression.

After 3 months, there was a statistically significant difference between the two groups: the biofeedback group had lower DHI scores, indicative of less self-perceived disability than the control group. The reduced DHI score in the experimental group was indicative of a reduction in the self-perception of the incapacitating effects on the quality of life caused by dizziness. This may have been produced by the fact that those who attended biofeedback were learning to regulate respiratory frequency, heart rate, and muscle tension parameters, and this had a positive impact on the physical, functional, and emotional components related to vertigo. Moreover, between biofeedback sessions, patients were also asked to practice improving their breathing, relaxing muscles, and managing their disorder. It is possible that this treatment produced a reduction of dizziness and DHI scores and an improvement in physiological parameters in the biofeedback group.

The differences between groups included both the DHI total score and all three sub-scales, although the greatest difference was the emotional component of the DHI. Furthermore, in the biofeedback group, the psycho-physiological parameters showed a reduction in the average values at three-month follow-up compared to the baseline. In particular, a reduction in the parameter relating to muscle tension, skin conductance, heart rate, and respiratory rate was observed, as well as lower maximal respiratory and heart rate. This study also demonstrated a reduction at three-month follow-up, not only in the average number of breaths but also in maximal respiratory and heart rates. During episodes of vertigo, patients showed an activation of the sympathetic nervous system with hyperventilation and an increase in their respiratory rate.

The positive results achieved in only a few sessions can be explained by the fact that the first sessions were dedicated to hyperventilation, as they focused on respiratory regularization, which in turn reduces nystagmus. Indeed, the short-term therapeutic goal of each biofeedback session was the self-regulation of breathing and muscle tension through breathing and muscle relaxation procedures. It should be highlighted that patients with vertigo not only have respiratory deficits due to hyperventilation, but also have incorrect postures due to muscle rigidity caused by vertigo and motor instability. In addition, patients drastically reduce any movement of their head and neck due to the fear of the onset and worsening of the vertiginous symptom. After training with biofeedback, patients acquired the ability to regulate their respiratory rate by learning to recognize the trigger factors of respiratory activation. In muscle relaxation exercises, an improvement in vestibular function was also observed through guided exercises for the head, shoulders, and face, performed with eyes closed.

Furthermore, for the reduction of muscle tension, the indications of Jacobson’s progressive relaxation protocol (Jacobson, 1987) were followed and, for each muscle band involved, the patients were asked to observe their own muscle tension on the biofeedback monitor, trying, thanks to the signal feedback, to reduce muscle tone by increasing progressive relaxation. These progressive relaxation exercises were then assigned as homework in order to improve awareness in subsequent sessions.

Biofeedback allowed patients to view their movements and muscle tension in real time while performing relaxation exercises. Patients in the biofeedback group therefore acquired greater control of some of the physiological parameters. Indeed, biofeedback is based on the concept that if people are informed of the variations of a physiological parameter of which they are not normally aware, they can learn to control it to some extent. When people reduce muscular tension, then EMG activity also reduces, with a return sound signal that decreases in frequency. As such, patients are ‘guided’ to relax and learn to self-control their state of tension through subsequent tests (Biondi et al., 2014). In the biofeedback group, a reduction in sympathetic autonomic activity and induction of a parasympathetic prevalence through relaxation were observed as well as reduction of anxiety in dizzy patients. Indeed, patients recognize the association between body indices (e.g., heart rate, muscle tension, electrodermal activity, etc.) and their internal state by learning to recognize the association between the latter and the emotional states that accompany them. After learning and recognizing this association, subjects voluntarily check their body indices by reducing sympathetic system activity and increasing the parasympathetic system.

### Limitations

There are some limitations we should acknowledge in this study. The first limitation regards the generalizability of our results. The sample was not very large, the recruitment number was not based on a sample size estimate, and the sample came from just one medical center. Moreover, the sample chosen was not an arbitrary choice, although neither was it an actual randomization. The second limitation concerns the absence of data on patient personality characteristics or other features that may have allowed us to obtain a more homogeneous sample (e.g., no preliminary assessments were made on the presence of any personality disorders that could aggravate the pathology in progress). The third limitation was that it was not possible to determine if the control group had any change in psycho-physiological parameters (only monitored in the biofeedback group), because these parameters were not measured for the controls. Finally, a further limitation of this study is that the mechanism through which biofeedback allowed a resolution of the vertigo symptoms is not currently known. In addition, several biofeedback protocols (EMG, HRV, and respiratory) were used, and it is unknown which protocol contributed most to the outcome. A reduction in the patient’s sympathetic activation and a reduction in anxiety can be hypothesized. However, further studies are needed to investigate the mechanism by which biofeedback works.

## Conclusions

While the use of biofeedback for stress management has long been known in the literature, few studies are currently available for the treatment of vestibular disorders (Liu et al., 2017; Necdet Ardıç et al., 2021). This study seems to demonstrate the therapeutic efficacy of biofeedback to treat patients with vestibular disorders in the routine of a medical center. Biofeedback can interrupt the vicious circles triggered by stress underlying the etiology of balance disorders, thus improving the quality of life of patients. Patients who are very frightened during acute phases and cannot manage the disease, can resume daily activities, acquiring greater self-efficacy and obtaining a reduction in vertigo-induced anxiety. Future research should integrate the use of biofeedback with other psychological treatment such as the cognitive behavioral therapy to increase the effectiveness of the treatment to investigate the psychological cognitive mechanisms underlying the vertiginous pathology.

## Data Availability

The data that support the findings of this study are available on request from the corresponding author.
