# Correction: Is Biofeedback for Vertigo Effective in Ordinary Medical Centers? A Controlled Trial in Northern Italy

**DOI:** 10.1007/s10484-023-09594-2

**Published:** 2023-06-13

**Authors:** Chiara Buizza, Elena Franco, Alberto Ghilardi

**Affiliations:** 1 Department of Clinical and Experimental Sciences, University of Brescia, Viale Europa 11, Brescia, Italy; 2 Medical Center San Francesco, Via Zadei 16, Brescia, Italy

**Correction to**: Applied Psychophysiology and Biofeedback

10.1007/s10484-023-09588-0

The original version of this article unfortunately contained a typo in co-author name.

The second author’s name should be spelled as Elena Franco instead it was published incorrectly as Eleno Franco.

The original article has been corrected.

